# Novel Per-Oral Cricopharyngotomy for Cricopharyngeal Bar: Feasibility Study With Emphasis on Technical Limitations

**DOI:** 10.7759/cureus.36663

**Published:** 2023-03-25

**Authors:** Karthik Pittala, Nolan Reinhart, Joseph A Sujka, Vic Velanovich, Christopher G DuCoin

**Affiliations:** 1 Department of Surgery, University of South Florida Morsani College of Medicine, Tampa, USA

**Keywords:** dysphagia, endoscopy, cricopharyngeal dysfunction, cricopharyngeal bar, c-poem

## Abstract

Per-oral endoscopic cricopharyngotomy (c-POEM) is a treatment for cricopharyngeal dysfunction, specifically cricopharyngeal bars (CPB). C-POEM differs from other endoscopic surgical procedures, such as per-oral endoscopic myotomy (POEM), gastric per-oral endoscopic myotomy (g-POEM), and Zenker per-oral endoscopic myotomy (z-POEM). We report three patients who underwent c-POEM for CPB, their clinical course, and outcomes. We underwent a single institution retrospective chart review of three patients who underwent c-POEM and their immediate postoperative course. These three patients represent all patients who underwent c-POEM. The operating surgeons were experienced endoscopists who regularly performed endoscopic myotomy. The three patients were female, over 50 years old, and presented with dysphagia secondary to the CPB. All three patients had perioperative complications consistent with esophageal leaks requiring prolonged hospital courses and recovery. All three patients had improved but persistent dysphagia up to nine months following the procedure. The results of this small case series exemplify the high rate of complications, specifically postoperative esophageal leak, when performing c-POEM for CPB. Thus, we stress caution and recommend against performing c-POEM for CPB.

## Introduction

Cricopharyngeal bars (CPBs) are relatively common but rarely cause symptoms, with only 5% of patients reporting bothersome complaints [1]. They are part of a spectrum of cricopharyngeal dysfunction, which includes Zenker’s diverticulum, cricopharyngeal achalasia, and neurologically induced swallowing dysfunction [2]. CPBs result from the hypertrophy, myositis, and fibrosis of the cricopharyngeal muscle, which reduces the maximal dimension of the upper esophageal sphincter, despite normal relaxation of the sphincter [3]. First-line therapy for CPB is esophageal dilation with a large bougie or balloon, with surgical myotomy reserved for cases intractable to dilation [4,5]. However, with the success of per-oral endoscopic myotomy (POEM) for Zenker’s diverticulum (z-POEM), per-oral endoscopic cricopharyngotomy (c-POEM) seems like a natural extension of the technique [6,7]. As our group has had several years of experience with POEM, including z-POEM, we felt that c-POEM should be offered to our patients. We report our experience with three patients who underwent c-POEM for CPB and their outcomes. This article was previously presented as a meeting abstract at the 2022 Society of American Gastrointestinal and Endoscopic Surgeons (SAGES) conference on March 16, 2022.

## Case presentation

Case 1

**Figure 1:**
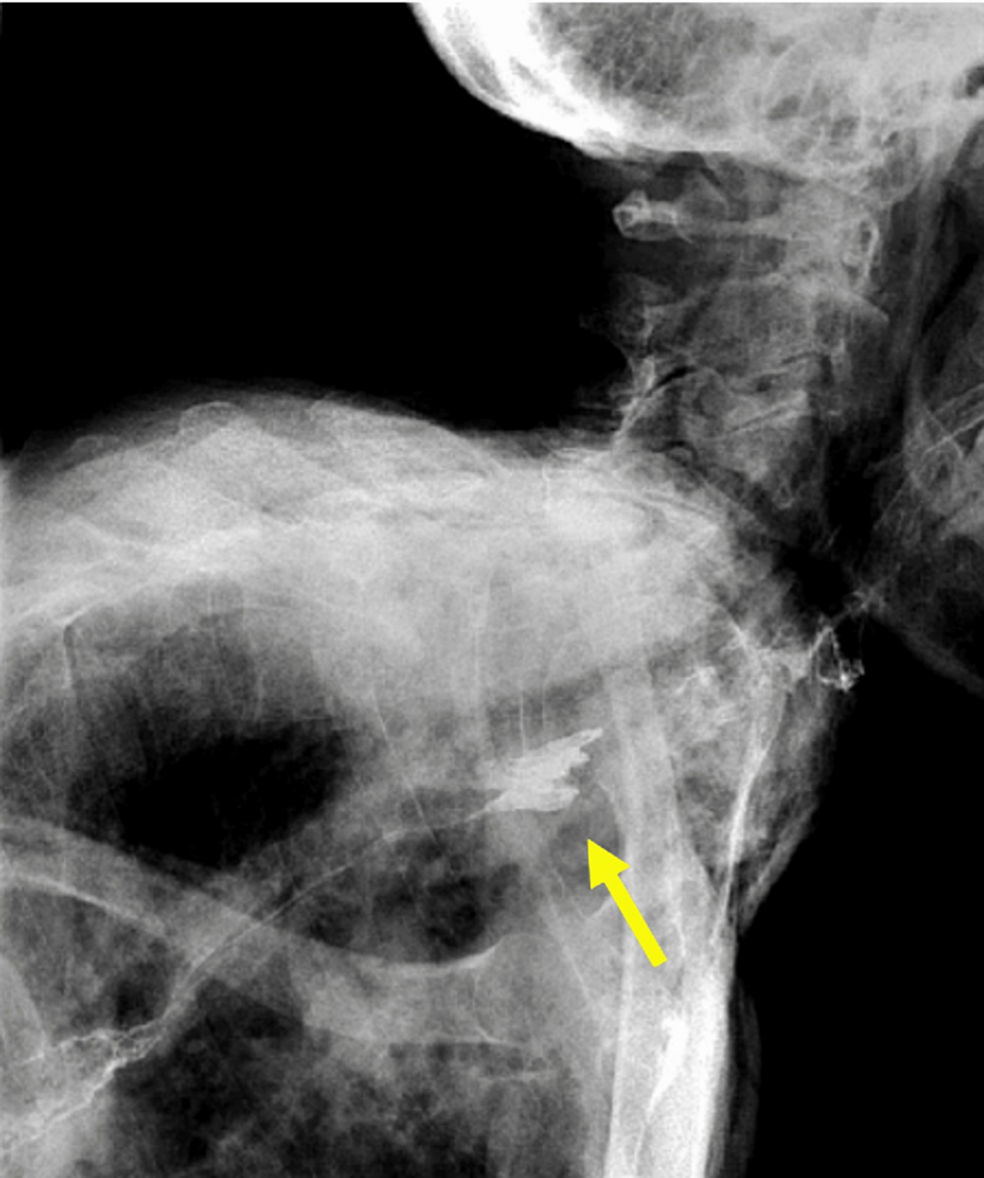
Case 1: Barium swallow study showing the presence of a cricopharyngeal bar.

An 83-year-old female with a past medical history of dysphagia presented for evaluation of a significant CPB. She had chronic complaints of difficulty swallowing pills and silent aspiration. On the esophagram, a 90% occluding CPB was noted (Figure 1). The patient underwent esophagogastroduodenoscopy (EGD) with findings of CPB at 15 cm from the incisors, noting a strong pulsation in the same location. As there was a concern for dysphagia lusoria, a CT scan of the neck and chest was performed, which ruled out the diagnosis of aberrant anatomy, the pulsation being attributed to the aortic arch.

**Figure 2:**
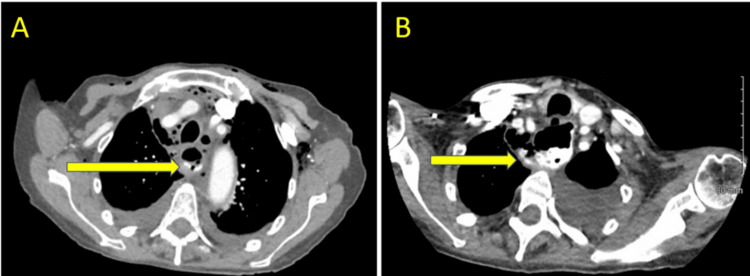
Case 1: CT scans showcasing esophageal leak with pleural effusion on (A) POD 3 and (B) POD 5. POD: Postoperative day.

Subsequently, the patient underwent c-POEM. A mucosotomy was created, a tunnel was formed, and the CPB was completely transected with triangular-tip knife electrocautery. It was noted that while the clips closed the mucosa, they seemed to be moving in a volatile manner in this location of the oropharynx. On postoperative day (POD) 2, the patient desaturated, and a CT scan noted an esophageal leak with a pleural effusion (Figure 2). Upper endoscopy revealed a small non-healing mucosotomy. The area was not amenable to clip closure; therefore, an endovac was placed, but a repeat swallow study showed a persistent leak.

Secondary to this leak, the patient developed a posterior mediastinal collection requiring rigid esophagostomy and left neck dissection with drainage of a posterior mediastinal abscess. The patient had a prolonged hospital course after drainage, managing these drains, and eventual discharge. She was discharged on POD 18, with drain removal scheduled on POD 29. She passed a swallow study and progressed to a regular diet, with complete recovery at her three-month follow-up. However, while able to tolerate liquids and a mechanical soft diet, she still had dysphagia with specific solids.

Case 2

**Figure 3:**
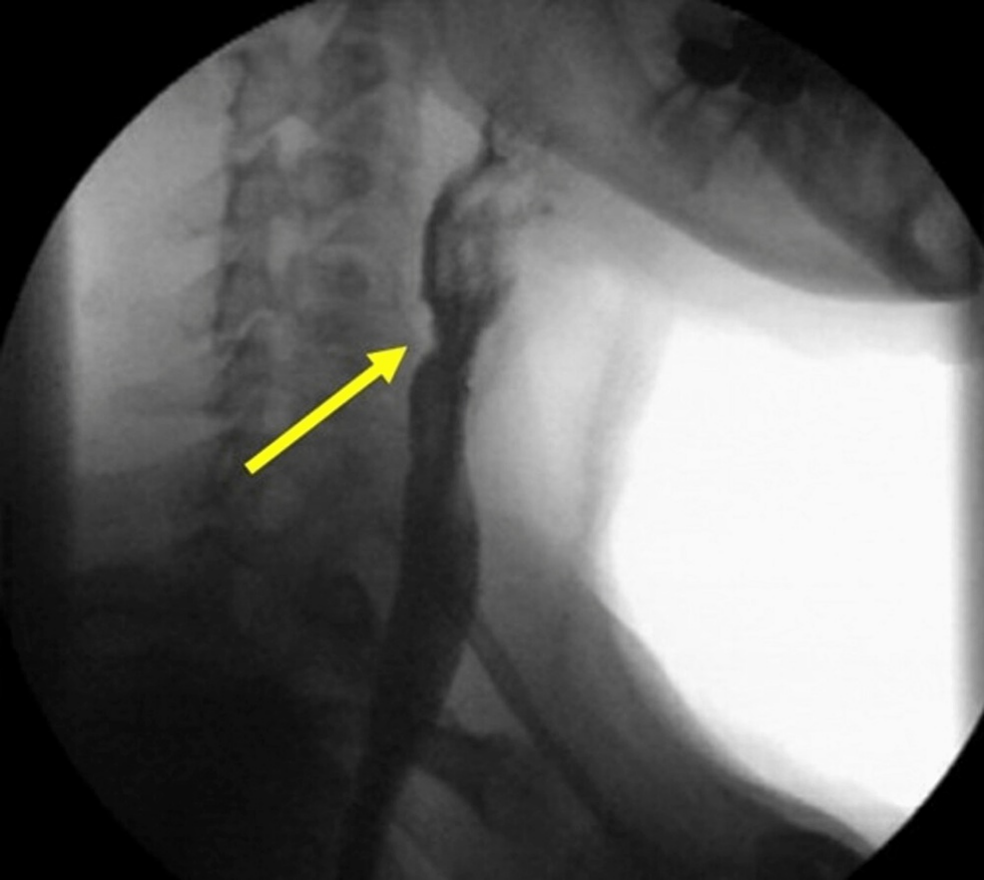
Case 2: Barium swallow study showing the presence of a cricopharyngeal bar.

A 59-year-old female with a past medical history of fibromyalgia, hiatal hernia, and hypothyroidism presented with dysphagia and globus symptoms. She was diagnosed with a CPB via barium swallow study and EGD, which showed a 50% occlusion (Figure 3). Over the previous 10 years, she had eight EGDs performed with attempts at dilation but no long-lasting symptomatic relief. Because of this, she was evaluated for c-POEM.

**Figure 4:**
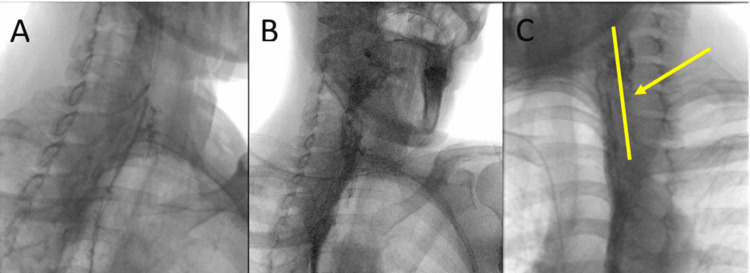
Case 2: Upper GI series showcasing esophageal leak on (A) POD 6, (B) POD 8, (C) POD 9.

During the procedure, a standard upper endoscope was used. The bar was found at 15 cm with no additional pathology noted. A longitudinal mucosotomy was made 2 cm superior to the bar, and the submucosal space was developed. During this case, a narrow working space and difficulty separating the mucosa from the bar made tunnel development difficult. This limited the working space in the tunnel for the endoscope, making deflecting the tip of the endoscope very constrained. Using the triangle-tipped electrocautery device, the muscle of the CPB was divided. At the completion of the myotomy, it was noted that the mucosotomy defect was large, consisting of about 40% circumference of the esophagus. We could not close the defect with the standard endoclips and needed to perform endoscopic suturing. On POD 2, the patient was found to have an esophageal leak on contrast esophagography. The decision was made to forgo additional endoscopic closure methods, and a nasogastric tube (NGT) was placed for drainage. The patient had a prolonged course of conservative management and was discharged home on POD 13 (Figure 4). After discharge, the patient still reported dysphagia. Due to continued dysphagia, she had undergone two subsequent esophageal dilations with Savary dilators, the last of which was 60 French. Her only continued complaint of note is dysphagia with solids.

Case 3

**Figure 5:**
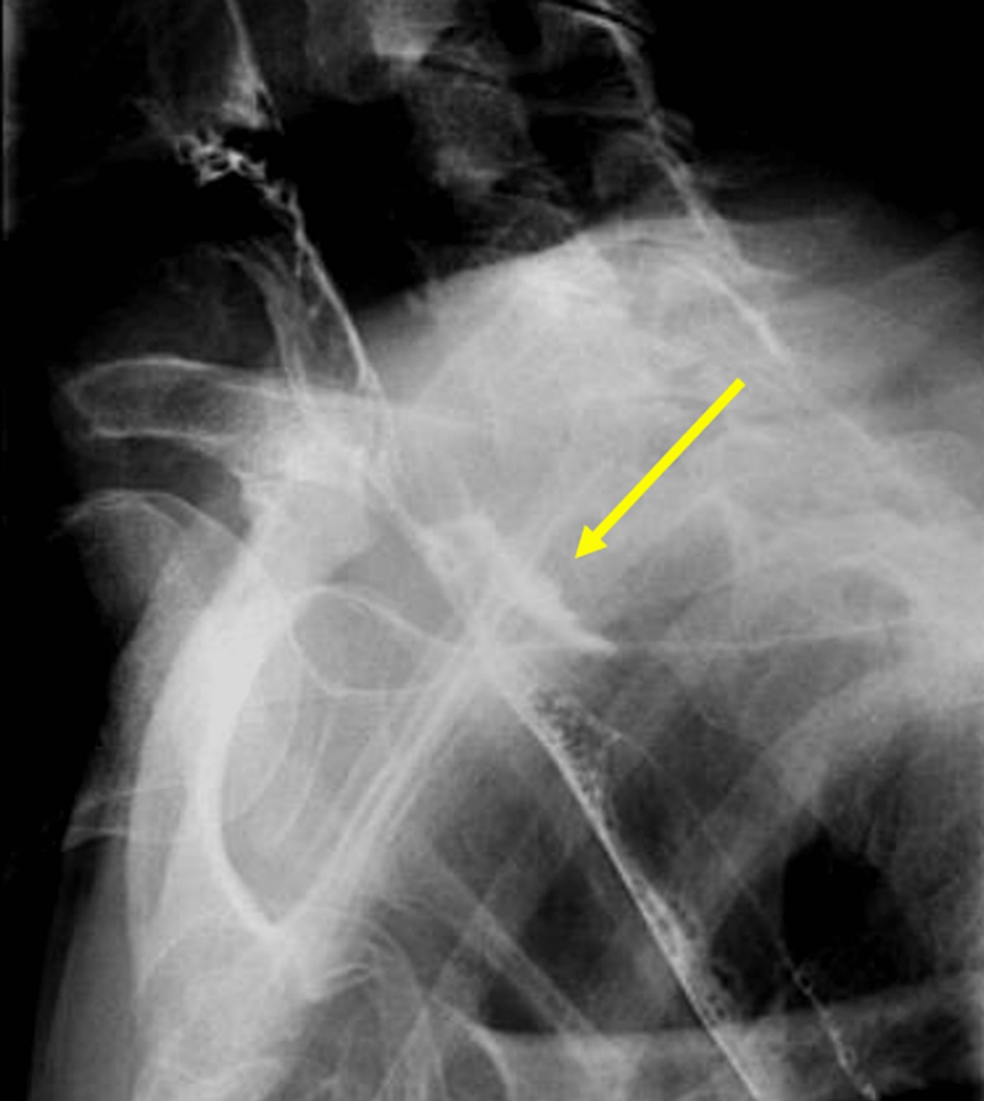
Case 3: Barium swallow study showcasing esophageal leak on POD 4. POD: Postoperative day.

A 68-year-old female presented with pharyngeal dysphagia. She had a past medical history of a hiatal hernia and coronary artery disease with a remote history of myocardial infarction, hypercholesterolemia, and hypertension. A barium Esophagram identified the presence of a large CPB, which did not improve with dilation. Therefore, she was evaluated for c-POEM. Again, using a standard upper endoscope, the bar was identified as essentially occupying the entire lumen of the hypopharynx at 90%. As with the other cases, the working space was severely constrained, limiting the amount of deflection possible. Nevertheless, a longitudinal mucosotomy was made on the posterior surface of the esophagus 2 cm superior to the bar, and the submucosal space developed. There was limited workspace and difficulty in developing the submucosal plane, and the tunnel could not be extended beyond the bar. We, therefore, chose to abort the procedure. The mucosotomy was closed with endoscopic clips. The next day, the patient underwent a cricopharyngotomy through an open left neck incision. Even after the myotomy was completed, the esophageal mucosa remained stenotic, as if the mucosa was fibrotic. An upper endoscope was passed into the hypopharynx, which confirmed that the esophageal narrowing was not alleviated with the myotomy. Using the existing mucosotomy and removing the endoclips to close the mucosotomy, a mucosal stricturoplasty was performed by dividing the esophageal mucosa across the fibrotic web longitudinally, then closing the resultant esophagotomy transversely. We elected to reinforce with a sternocleidomastoid muscle flap. Despite this, the patient developed an esophageal leak through the cervical incision on POD 4 (Figure 5). This was managed conservatively with the eventual healing of the leak. The patient, however, had persistent dysphagia. At her three-month follow-up, she underwent upper endoscopy with Savary dilation. The endoscopy demonstrated a “keyhole” deformity consistent with a myotomy with stricturoplasty, but there was a persistent ridge suggestive of a residual bar. After dilation, contrast esophagography demonstrated the resolution of the bar. Although she is tolerating a regular diet, she still reports dysphagia.

## Discussion

Historically, CPB has been treated with dilation, botulinum toxin injection, or open surgery [8]. A study by Patel BJ et al. showed that Savary dilation is both safe and effective, with most patients (81%) free of symptoms at six months [4]. In another study, Brigand C et al. reported similar outcomes for balloon dilation [9]. Surgical cricopharyngotomy is associated with significant morbidity [9]. Different studies report a complication rate varying from 3.7% to 15.8% and 1.6% mortality in larger series for transcervical cricopharyngeal myotomy (CPM) [10,11]. A review of the literature has shown mixed results in terms of complication rates and success when attempting c-POEM. Table 1 details complication instances in three studies that compiled outcome data on c-POEM. While the complication rate in botulinum injection is 9%, there is a concomitant high rate of recurrence and the added risk of worsened dysphagia in certain cases [12]. A surgical option with less morbidity would be a welcomed addition to the treatment armamentarium for CPB. Huntley C et al. compared 41 endoscopic procedures to 37 open cases [10]. They demonstrated endoscopic cricopharyngotomy yielded a similar resolution of dysphagia symptoms to open CPM and had a significantly lower operative time, with no difference in complication rates [10]. It is unclear whether the method of cricopharyngotomy performed using a laser vs. triangle-tipped electrocautery is the reason for improved outcomes. It is also somewhat unclear the exact method utilized by the authors for cricopharyngotomy [10]. The results found by Huntley C et al. may be due to the volume of patients [10]. Outcomes for more recent series on c-POEM are less encouraging. Elmunzer BJ et al. presented three cases of the CPB where c-POEM was successfully completed [11]. However, post-op leakage occurred in two of the three cases leading to hypopharyngeal edema, which was treated with conservative management and prolonged the patient’s hospitalization by several days [11]. This is similar to our experience, and while Elmunzer BJ et al. [11] are more optimistic in their evaluation of c-POEM, we do not share this optimism. With our findings of 100% complication rate and only mild improvement in dysphagia symptoms after c-POEM, we have ceased offering this procedure to our patients.

**Table 1:** Review of current literature on c-POEM treatment outcomes and complications. c-POEM: Per-oral endoscopic cricopharyngotomy.

Study	Year	Treatment	Arms	Major Outcomes	Dysphagia Outcomes	Complications	Critical Points
Huntley C et al. [10]	2017	41 Endoscopic; 22 Carbon Dioxide laser; 19 Diode laser	38 Open, Left-sided transcervical approach	Case length, time to oral intake, hospital length of stay, length of follow-up, and changes to dysphagia	Endoscopic 40/41 (98% improve dysphagia); Open 30/38 (79% improve dysphagia)	Endoscopic 2/41 (5%), Open 5/38 (13%)	No validated dysphagia questionnaire used; Short follow-up; Decreased length of case, only significant outcome
Elmunzer BJ et al. [11]	2020	3 Endoscopic		Resolution of dysphagia, tolerance of an unrestricted diet	Endoscopic 3/3 (100% improved dysphagia)	Endoscopic 2/3 (66%); prolonged hospital stay due to edema	No validated dysphagia questionnaire used; Follow-up not reported; 66% of patients had complications
Pittala K et al.	Current Case Series	3 Endoscopic		Resolution of dysphagia, tolerance of unrestricted diet	Endoscopic 0/3 (0% reported improved dysphagia)	Endoscopic 3/3 (100%); Prolonged hospital stay, esophageal leak	No validated dysphagia questionnaire used; Follow-up ongoing; 100% complications

In our experience with this technique, a few common problems have been observed, including trouble with the mucosotomy tunnel formation and closure due to a tight working space. The c-POEM mucosotomy proved more troublesome than other POEM procedures, including z-POEM, because the CPB itself occupies so much of the luminal space. Anatomic limitations are also present, making this procedure more difficult. First, the hypopharynx does not distend well and is very dynamic in its movement, making creating a stable working environment difficult. Using a standard endoscope of 10.8 mm in diameter very much constrains the ability to deflect the tip for proper positioning of the endoscopic instruments. Secondly, due to the nature of the CPB being convex and into the lumen of the esophagus, the resultant mucosotomy is longer and wider than with other POEM procedures. Thirdly, the submucosal tunnel is shorter than similar procedures. Thus, there is a higher risk of a full-thickness leak as the tunnel cannot collapse, allowing the mucosal layer to seal with the non-cut muscle. Fourthly, the cricopharyngeal muscle is a single muscle of circular fibers, lacking the longitudinal fibers of the more distal esophagus, meaning a full-thickness myotomy will result in complete disruption of the luminal wall. In addition, the fibrotic nature of the CPB makes it more of a mechanical obstruction than an obstruction due to muscle spasms [3]. One other issue is that even after the division of the CPB, there still may remain a fibrotic ridge which can be associated with dysphagia. Some possible solutions to these technical limitations could be using a smaller diameter endoscope, allowing for more maneuverability in tight spaces. Alternative methods for closing the mucosotomy, such as fibrin glue or endoscopic suturing, can be utilized. In one of our cases, we used an endoscopic suture for the closure of a difficult mucosotomy. This has been noted in the literature for use during z-POEM and maybe another possibility for c-POEM closures if clips are unsuccessful [11].

## Conclusions

We feel that there are technical and pathophysiological limitations to the treatment of CPB with c-POEM. Due to the results of this study, we believe that the first-line treatment for CPB should be dilation, with our choice being Savary dilation. Only after failure of repeated dilation should myotomy be considered. If considering c-POEM, one should be prepared for the technical limitations and anatomical challenges of the procedure. Currently, the authors do not feel that c-POEM should be recommended as the first-line treatment of CPB.
